# A Simulation Model of Neural Activity During Hand Reaching Movement

**DOI:** 10.32598/bcn.9.10.390

**Published:** 2020-01-01

**Authors:** Sohrab Saberi Moghadam, Mahsa Behroozi

**Affiliations:** 1. Faculty of Engineering Modern Technologies, Amol University of Special Modern Technologies, Amol, Iran.; 2. Department of Physiology, Faculty of Biology and Medicine, University of Lausanne, Lausanne, Switzerland.; 3. Neuroscience & Neuroengineering Research Lab., Department of Biomedical Engineering, School of Electrical Engineering, Iran University of Science and Technology (IUST), Tehran, Iran.

**Keywords:** Poisson model of neural activity, Neural variability, Hand movement, Cortex

## Abstract

**Introduction:**

The neural response is a noisy random process. The neural response to a sensory stimulus is completely equivalent to a list of spike times in the spike train. In previous studies, decreased neuronal response variability was observed in the cortex’s various areas during motor preparatory in reaching tasks. The reasons for the reduction in Neural Variability (NV) are unclear. It could be influenced by an increased firing rate, or it could result from the intrinsic characteristic of cells during the Reaction Time (RT).

**Methods:**

A neural response function with an underlying deterministic instantaneous firing rate signal and a random Poisson process spike generator was simulated in this research. Neural stimulation could help us understand the relationships between the complex data structures of cortical activities and their stability in detail during motor intention in arm-reaching tasks.

**Results:**

Our measurements indicated a similar pattern of results to the cortex, a sharp reduction of the normalized variance of simulated spike trains across all trials. We also observed a reverse relationship between activity and normalized variance.

**Conclusion:**

The present study findings could be applied to neural engineering and brain-machine interfaces for controlling external devices, like the movement of a robot arm.

## Highlights

The simulation of neural spike trains in the cerebral cortex was conducted during a reach-to-grasp task performed by a monkey.

The definition of neural mechanisms and trial-to-trial variability in the execution of movements by the simulation model of spike trains as neural activity; they were measured by normalized variance as neural variability.

The reduction of simulated neural variability, compared to similar behavior in the real variation of cortical activity after target onset or before the movement was observed.

## Plain Language Summary

The brain activities are nonidentical in repeated trials during the motor preparation and hand movements. This variation of cortical neural responses could result from the internal and external properties of individual neurons or neural populations. The stimulus onset reduces neural cortical variability during the hand movement. If this reduction is coordinated to variability in the sensorimotor neural population, then the observational error movement should have represented low values. To understand the mechanism of reduction, the neural response has been simulated as a random Poisson process spike generator with or without noise. The simulation model demonstrated a decrease in variability of simulated neural responses with noise after the target onset; however, no changes of activity was observed in the absence of noise. The simulation of this mechanism provides a great opportunity for a better understanding of the neurophysiological substrate of the neurodegenerative and mental conditions. Applying neural variability in predicting hand movements control could support brain-machine interfaces for stroke patients’ paralysis and even neuro-feedback therapy. Controlling hand movement could be modeled in a closed-loop neural circuit with added noise and neural variability.

## 1. Introduction

**Figure 1.**
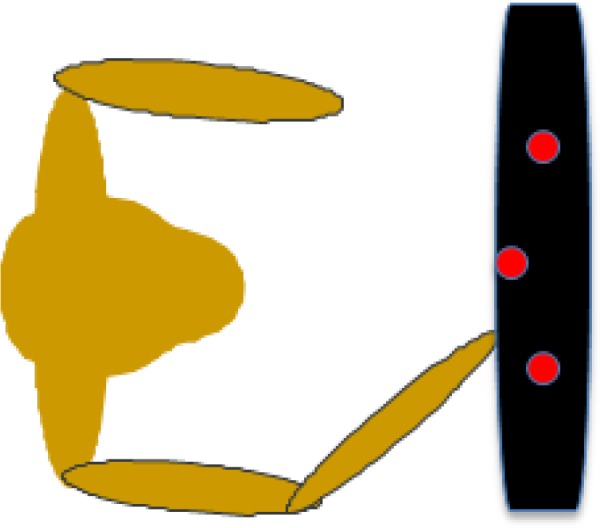
The monkey was trained for the arm-reaching task After training, long-term neural recordings in behaving animals were collected for pre-processing.

Cortical cells receive inputs from many sources, and the distribution of post-synaptic potentials is Poisson-like (Anderson, Carandini, & Ferster, 2000; Carandini, 2004). The brain activity is inherently variable, as its output in the form of motor behavior. The response of cortical neurons to an identical stimulus repeated over time exhibits large variability in spiking activity. The Neural Variability (NV) is characterized by the ratio of variance to the mean value of spike density function across repeated trials (Churchland et al., 2010; Shadlen & Newsome, 1998; Teich, Heneghan, Lowen, Ozaki, & Kaplan, 1997). The pure Poisson distribution with independent information follows the firing rate between spikes; accordingly, the measured NV equals unity, and for contaminated neural spike activity by additive noise, the latter exceeding unity (Geisler et al., 2005; Mazurek & Shadlen, 2002; Shadlen & Newsome, 1998). Above all the stimulus-driven neural responses, a noisy Poisson activity reduces the neural variability across cortical areas (Churchland et al., 2010). The exponential Poisson distribution of the neural response would be formed to a more regular compact pattern by increasing the firing rate and the refractoriness together (Maimon & Assad, 2009). During delayed reach-task, an increase in the firing rate after the target presentation would present a sharp decrease in variability during motor intention in the dorsal premotor cortex of monkey (Churchland, Byron, Ryu, Santhanam, & Shenoy, 2006). Briefly, researchers designed a behavioral tasks for monkeys. Monkeys were trained to sit in a primate chair to perform a set of arm-reaching tasks. Every task consisted of several trials, and each trial started with a stimulus onset (e.g. central spot on the screen) and ended with a juice reward if successful (Figure 1).

After designing a behavioral task and training the monkeys for a specific experiment, the neural data could be recorded. There are several methods for recording neural activity. For instance, the extracellular activity could be recorded with electrodes (glass-coated tungsten-platinum fibers; 1–2 M_impedance at 1 kHz) using a Thomas Recording system.

**Figure 2.**
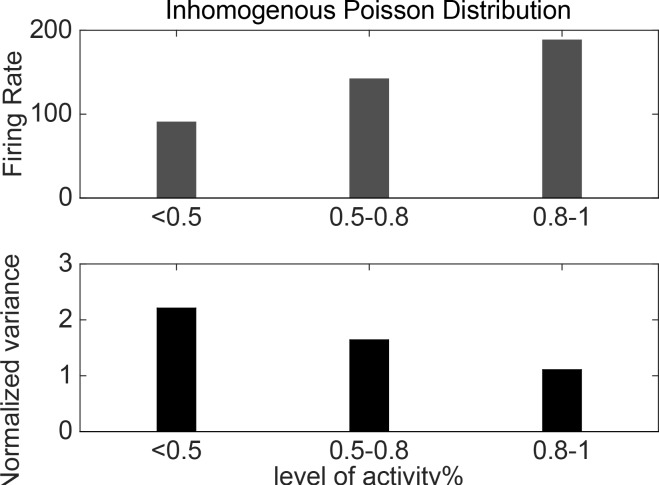
The different levels of maximum values (spike count from random Poisson spike trains) for each associated neural variability across trials

In the current study, the neural Poisson distribution time-locked to the target onset was simulated. Indeed, the struggle was the quantification of a relationship between the simulated neural Poisson spike process and the measured neural variability. There are several rate coding methods to measure the firing rate of single cells or population activity (Figure 2). The endogenous activity of a time-varying cell, as a stimulus-response, is a Spike Density Function (SDF). SDF for each movement direction and cell have been calculated. Initially, operating spike timing alignments were performed to the onset of the hand movement periods or the target presentation in trials, respectively. Secondly, spikes were replaced by a Gaussian function with a width (SD) of 30 ms. Consequently, a full area of 1.a continuous signal (firing rate) was observed after the normalization (considering the trial numbers) to produce the SDF for each cell and movement conditions. To compute the mean firing rate, we also averaged SDFs across trials for each movement direction (Saberi-Moghadam, Ferrari-Toniolo, Ferraina, Caminiti, & Battaglia-Mayer, 2016).

## 2. Methods

The first and second-order moment statistics were applied to estimate the mean and variance parameters of neuronal responses. The NV, i.e. the time function of trial-to-trial variability, is defined as the variance-to-mean ratio of spike density function across trials. This method is proposed by some scholars (Churchland, Afshar, & Shenoy, 2006) with the following Formul 1:

1. $$\mathrm{NV}(t)=k\times \frac{ɛ+\mathrm{Var}\left(SDF\left(t\right)\right)}{k{ɛ}^{\prime }+Mean\left(SDF\left(t\right)\right)}$$

*Where ε=ε′=0.01 and k=0.1.*

To prevent the ratio of NV from becoming zero in the dominator in some cases, the constant value was added. Based on the assumption that neural activity is commonly generated as the Poisson process, the mean values of the firing activity and variance are identical; thus, NV is unity. This value of fraction could be changed depending on the internal and external states of the individual and population neurons.

The neural activity of each neuron in contact with the neural network in various areas of the cortex exhibits chaotic patterns; it implies the intrinsic and extrinsic characteristics of that cell. Therefore, to detect a cell’s characteristics for repeated trials (in random responses to the same stimulus), firing rates were simulated (equal to SDF) in two types of pure Poisson distribution of firing rates and pure Poisson activities by adding a random noise (noisy Poisson distribution). The aforementioned random noise contains random numbers that follow a Gaussian distribution. The pure Poisson distribution is a set of Poisson random numbers generating random numbers from the Poisson distribution with an average number of occurrences μ over a period. Poisson distribution is an event with a small probability of occurrence and a large number of independent trials taking place. The obtained NV has revealed different behaviors for each group. To use the Poisson spike generator in a simulation, a model neuron is required. The probability of spikes for a Poisson distribution is as Formul 2:

2. $$P\left(X=k\right)=\left(e^{-\mu }\times \mu^{k}\right)/k!$$

Where is the average number of spikes per intervals, e is the Euler’s number, and the parameter is a Poisson random variable that takes non-negative discrete values 0, 1, 2, and so on.

## 3. Results

To determine the relation between NV and firing rates, the mean firing rate of simulated activity was firstly computed. Then, the normalized value of neural response was found through calculating the maximum value of the firing rate. Next, the normalized value of the amplitude of the neural response was divided into three levels, i.e. the maximum value of the response, (0.8%–1%) of maximum value, (0.5%–0.8%) of maximum, and <50%, and finally, the distribution value of NV related to each level was found.

There was a reverse relationship between the firing rate and normalized variance. The neural variability was declined by an increased firing rate.

**Figure 3.**
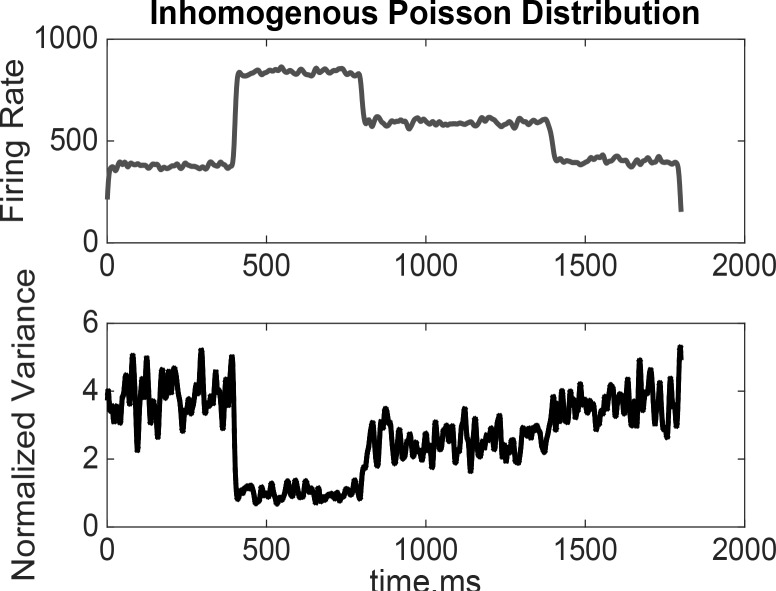
The time course of the simulated spike train (firing rate) and variability in an inhomogeneous Poisson distribution NV, aligned to the target presentation, was declined by the change in the population neural activity represented in the form of population spike density function or firing rate.

Previous studies demonstrated the temporal evolution NV (±, computed across individual cells and target directions) significantly declined in the frontal areas after the target presentation or before the movement onset. To overcome unreliable statistical results across repeated trials, selected neurons and all directions were pooled. The neural signal and neural variability were averaged across all trials and directions. This strength of NV reduction in the frontal lobe could be a result of increased activity only within specific time windows that depends on the considered area. In our study, the simulated level of neural activity was changing during the time course of behavior (Figure 3). The obtained results suggested the reduction of variation in time windows. It was confirmed that neural variability is a time course of neural activity to predict or code events.

**Figure 4.**
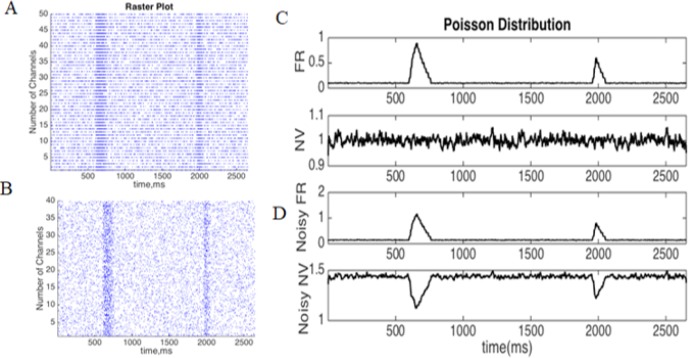
A Poisson model of a spike generator for 100 trials and 1000ms A: A raster plot of pure Poisson; B: Noisy distribution; C: Pure Poisson of the Firing Rate (FR), and the relevant neural variability. There was no change of neural variability (close to unity) even after a rise in the firing rate; D: Noise-contaminated firing rate, and the relevant neural variability. In this case, the NV increased the odds of changes after the jumps of the firing rates.

The simulation in Figure 4 illustrates the neural activity and variability for a neuron with Poisson spiking statistics. An inhomogeneous Poisson model (time-varying) was used to generate spike trains for 100 trials and 1ms duration, which we presented in the raster plots of 50 trials in Figure 4. A and 40 trials in Figure 4 B. The pure Poisson distribution of spike activities indicated linear effects between the variance and mean values of the firing rate, leading to a neural variability equal to one (Figures 4 A & C). If the simulated neural response was contaminated by the random noise with a random poison distribution, the nonlinear effects between the mean and variance cause the NV to become greater than one (Figures 4 B & D). The NV is sensitive to small changes in contaminated signals, and it decreases by increased neural activity in a noisy Poisson distribution. This decline results in the change of the activity and the internal states of neurons in the population level of neural networks.

**Figure 5.**
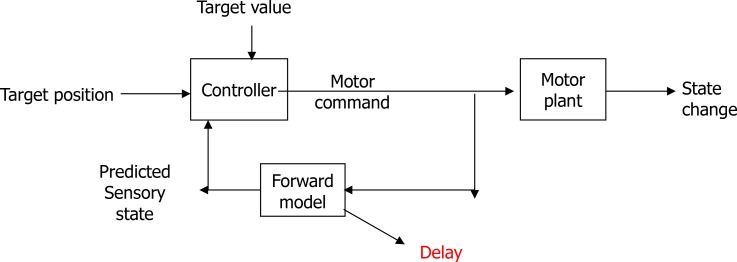
The block diagram of a closed-loop neural circuit of the motor planning cortex

Figure 5 illustrates a closed-loop of neural circuits of a motor plan. In the block diagram, the neural variability can be supposed as a disturbance; the motor cortex in the spiking neural network controller controls the complex motor information, and the output of motor command decodes the motor intention.

The copy of the movement was compared with electrical stimulation (input of a closed-loop system). The neural variability method could be applied in the brain-machine interfaces to support neurodegenerative diseases, like optic ataxia in human and animal research studies, to obtain a better understanding of the performance of this closed-loop neural circuits in the cortex, a mutual connection between the motor and the parietal cortex.

## 4. Discussion

No single study was performed on simulated neural variability to characterize the role of the noise in the brain. These study findings suggested that the neural variability, as an internal-external state of noise in the brain, was a function of modulations of trial-to-trial firing rate variability on the single and population level in the parietal, dorsal premotor, and motor cortices. In real data, the across-trial variability of firing rates through the normalized variance was first measured. In all investigated frontal and parietal areas, after the target presentation, a significant decline of NV was observed.

The neural variability is a disturbance or noise (target value) in this system and sensory feedback (forward model) to compare the changing status (movement) and the desired motor plan (target position). The closed-loop system in the motor cortex controls the motor plan.

To assess the dependency between the activity and the temporal or non-temporal pattern variability across repeated trials, spike trains designed through a random process were simulated; it generated pure Poisson distribution with no change of variability. By adding the noise to signal, a normalized variance was increased across contaminated trials. Moreover, the significant increment of the averaged noisy activity (by supposing time-locked to the stimulus onset) appeared a predominant impact on relative and absolute refractory periods of neuronal responses and a sharp decline of normalized variance on time patterns.

## 5. Conclousion

The neural variability associated with the composition of a motor plan described the task events and the behavioral performance of the animal in a satisfactory way. Moreover, there was a strong correlation between the neural and movement variability where the NV could predict the temporal evolution of movement planning and behaviors in humans. However, the mechanisms underlying the temporal variability of the neural planning in the human motor cortex and its characteristic changes in mental disorders could be related to the neurophysiological substrate of the disorder. By improving this method, as a scanner of the neural firing rate in the cortex, other forms of bio-signals in the other areas of the brain might also have potentials as a predictor of the behavior. These results could be related to the neurophysiological substrate of the disorder. To study this active inhibition notion, active task involvement is necessary to imply an interactive task performance. By improving the therapy with the suggestions mentioned above, other forms of neuro-feedbacks might also have potentials as a treatment for Attention-Deficit Hyperactivity Disorder (ADHD).
